# A Cardiac Amyloidosis Presentation: Atrial Mass Versus Thrombus

**DOI:** 10.7759/cureus.11944

**Published:** 2020-12-07

**Authors:** Saheba Nanda, Alex Gyftopoulos, Naomi Hardy, Allen P Burke, Robert TD Chow

**Affiliations:** 1 College of Medicine, American University of Antigua, Coolidge, ATG; 2 Department of Medicine, University of Maryland Medical Center, Baltimore, USA; 3 Department of Pathology, University of Maryland Medical Center, Baltimore, USA

**Keywords:** atrial thrombus, atrial myxoma, cardiac amyloidosis, transthoracic echocardiogram, transthyretin, cardiac thrombi, cardiac tumors, cardiac echo, transthyretin amyloid cardiomyopathy, amyloid plaques

## Abstract

Cardiac neoplasms are a rare finding of which a cardiac myxoma is the most commonly encountered. Therefore, a density identified in the left atrium commonly leads to the presumptive diagnosis of an atrial myxoma. However, other pathologies, such as atrial thrombi, can mimic in clinical presentation and appearance to a myxoma. Clinically, these pathologies may lead to obstructive symptoms such as syncope, palpitations, or sudden cardiac death. At present, echocardiography, magnetic resonance imaging, or computed tomography can be used to identify such masses, but fall short of identifying the primary cause. The management of atrial thrombi is not yet fully understood and definite recommendations have not been established. We present a case of an 87-year-old man complaining of syncopal episodes found to be secondary to an incidental intracardiac density resulting from age-related amyloidosis.

## Introduction

Atrial masses are often incidental findings discovered on cardiac imaging with an incidence of 200 in 1,000,000 cases [1]. Densities appreciated in the left atrium are presumptively diagnosed as atrial myxomas, which is the most common primary cardiac tumor found in the left atrium [2]. Due to the complexities of presentation and challenges inherent in obtaining pathologic confirmation, atrial masses are often difficult to diagnose and treat. An atrial thrombus can commonly mimic a myxoma in clinical presentation, appearance, and location on echocardiography [3,4]. Although numerous conditions may predispose an individual to atrial thrombi, cardiac amyloidosis is a common cause in elderly males [5].

Cardiac amyloidosis is relatively uncommon and has an incidence of 6.1 - 10.5 per million people per year with 1200 - 3000 new cases every year [6]. Dependent on the type of protein deposited, amyloidosis can be further classified into either primary (AL) or secondary type (AA). Cardiac involvement, observed in AL or AA subtypes, can lead to arrhythmias, congestive heart failure, hypertension, hypotension, and thrombi [7,8]. An autopsy study evaluating intracardiac masses in patients with and without a history of amyloidosis revealed a cardiac thrombus in 33% versus 0%, respectively [5]. In the elderly population, non-mutated wild type transthyretin (ATTRwt) accumulation may result in the formation of an atrial thrombus, which can be clinically misdiagnosed for an atrial myxoma.

At present, echocardiography is the primary diagnostic modality that can be used to identify atrial densities, but it falls short of identifying the primary cause [9]. Furthermore, there is limited literature on the use of anticoagulation for an atrial density identified as a thrombus. We present a case of an elderly man who presented with syncope and was found to have an incidental intracardiac thrombus secondary to ATTRwt amyloidosis.

## Case presentation

An 87-year-old man with a known past medical history of nonischemic New York Heart Association (NYHA) class 1 heart failure and stage 1 chronic kidney disease presented to the emergency department after experiencing a syncopal episode at home. The patient lost consciousness upon rising from his couch and denied any trauma from the fall. Prior to this episode, the patient was in his normal state of health and had been taking his medications as prescribed. His medication regimen included metoprolol 25 mg twice daily, amlodipine 5 mg once daily, valsartan 160 mg once daily, aspirin 81 mg once daily, glipizide 5 mg twice daily, and tamsulosin 0.4 mg once daily. The patient’s syncopal episode was not witnessed; he denied any associated seizure-like symptoms such as tongue biting, jerking movements, or urinary/fecal incontinence. The patient denied any prior similar episodes. He did not endorse any palpitations, vertigo, diaphoresis, nausea, or chest pain preceding this syncopal episode. He stated that the dosages of his antihypertensive medications were increased after a routine primary care visit one month prior. Vital signs upon arriving at the emergency department included a temperature of 36.4 °C, blood pressure of 110/55 mmHg without transient orthostatic changes, heart rate of 47 beats per minute, and respiratory rate of 17 breaths per minute. Auscultatory findings were significant for a loud S1 and a faint early diastolic murmur. No signs of left ventricular failure were present such as elevated jugular venous distention, lower extremity edema, or fine scattered crackles. Physical examination was otherwise unremarkable. 

The patient received intravenous fluid resuscitation. Electrocardiogram (ECG) showed sinus bradycardia without ischemic findings. Computerized tomography (CT) of the head and neck revealed no acute abnormalities, and a chest radiograph revealed cardiomegaly with no pulmonary congestion or infiltrates. A basic metabolic panel showed an acute kidney injury with an elevated blood urea nitrogen (BUN) and creatinine. Troponin I was elevated at 0.355 ng/mL which was thought to be secondary to the patient’s hypotension and chronic kidney disease. He was subsequently admitted for further evaluation.

**Figure 1:**
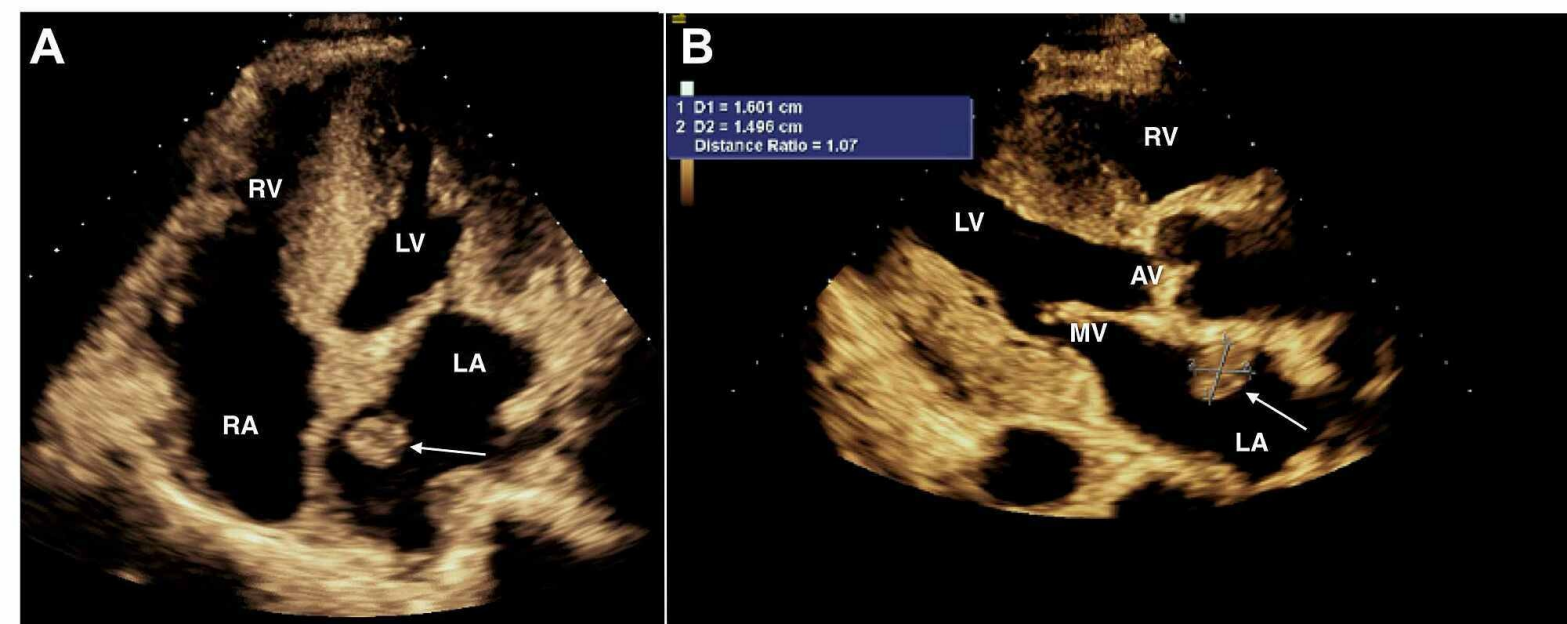
Transthoracic echocardiogram parasternal long axis and apical four-chamber view (A) Transthoracic echocardiogram apical four-chamber view revealing left atrial echocardiogram density denoted with a white arrow; (B) transthoracic echocardiogram parasternal long-axis view revealing 1.49 × 1.61 cm^2^ left atrial pedunculated echodensity, attached to the posterior wall of the left atrium denoted with the white arrow. LA: left atrium, LV: left ventricle, RA: right atrium, RV: right ventricle, MV: mitral valve, AV: aortic valve.

Due to the notable auscultatory findings, the patient underwent transthoracic echocardiography (TTE) that showed moderate concentric left ventricular hypertrophy with reduced systolic function, left ventricular ejection fraction of 45% without diastolic dysfunction, and a 1.49 × 1.61 cm^2^ mobile pedunculated echodensity in the left atrium attached to the interatrial septum, raising concern for an atrial myxoma (Figure 1).

**Figure 2:**
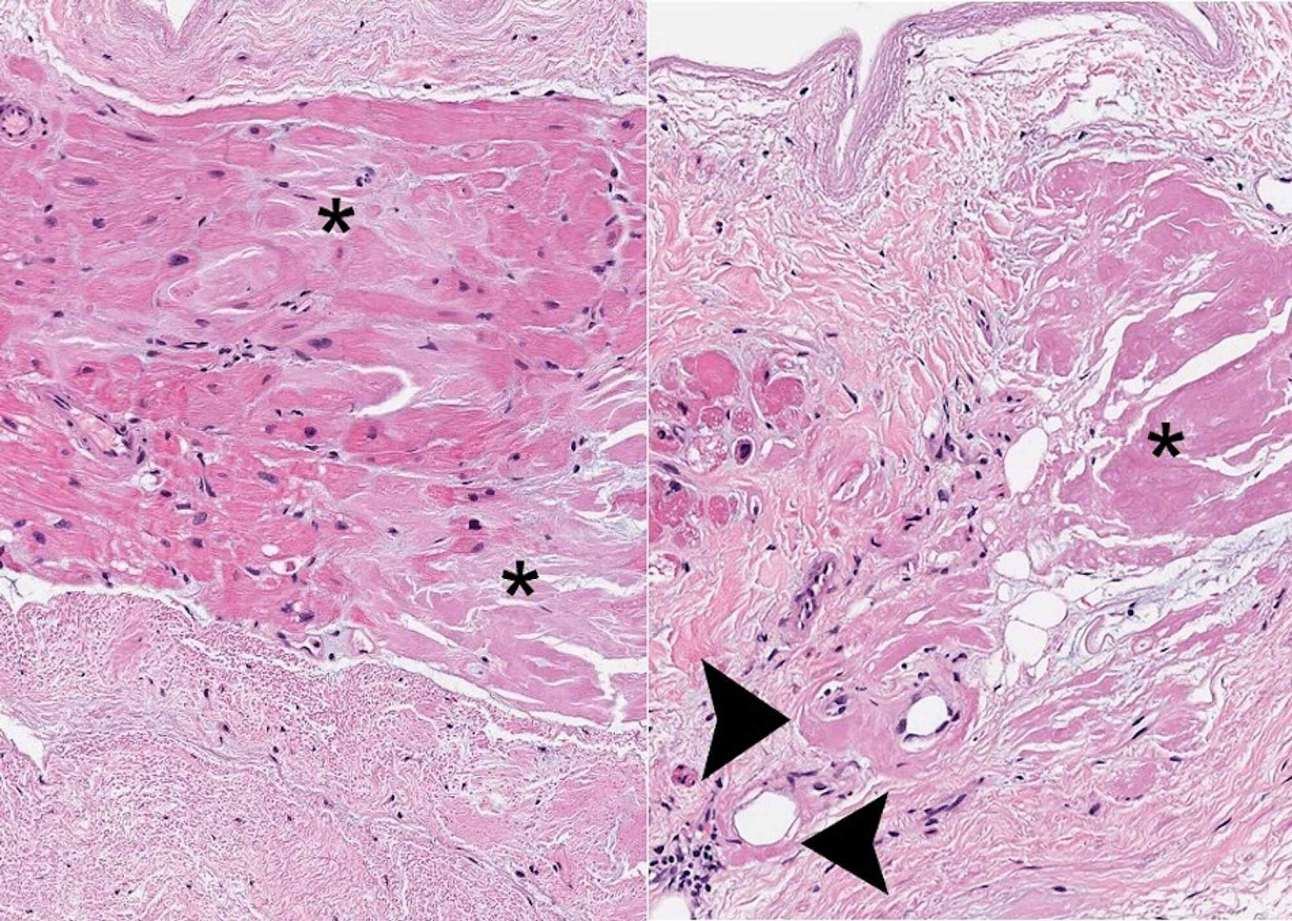
Histological evaluation of atrial mass Histopathological analysis of the excised left atrial thrombus. An endomyocardial biopsy demonstrates extensive extracellular deposition of amorphous eosinophilic material in the myocardial interstitium (asterisks) and also present perivascularly (arrowheads). H&E, 100×.

It was noted at this time, due to the biventricular hypertrophy and history of chronic kidney disease, cardiac amyloidosis was considered as a possible differential diagnosis. The patient was subsequently taken to the operating room for excision of his left atrial mass by cardiothoracic surgery. The operation concluded uneventfully, and the excised intra-atrial mass, surprisingly, was revealed to be a thrombus adherent to myocardial tissue. The atrial thrombus was then sent to pathology for further analysis. The histopathological report revealed that the mass was not a myxoma, but instead, a thrombus adherent to the myocardium. Histology of the adherent myocardium showed amorphous eosinophilic material (Figure 2), which was subsequently stained positive for Congo red stain indicating cardiac amyloid. The serum protein electrophoresis (SPEP), urine protein electrophoresis (UPEP), serum-free light chains, and amyloid subtype analysis confirmed non-mutated wild type transthyretin (ATTRwt), an age-related amyloidosis.

## Discussion

The imaging modalities used to diagnose cardiac masses found in the left atrium include an echocardiogram, both transthoracic and transesophageal. These modalities can characterize masses based on their size, morphology, extension, hemodynamic effects, and site of attachment [3]. Despite these measures, imaging is often limited due to artifacts that can be mistaken for multiple pathologies due to their morphological similarities, or in patients with poor acoustic windows [3,4]. To further investigate these masses, magnetic resonance imaging (MRI) and CT of the heart are often performed. Differential diagnoses for a mass found in the left atrium include myxoma, thrombus, sarcoma, lipoma, fibroma, and metastases. In the elderly ATTRwt, non-mutated wild-type transthyretin, amyloid deposits are frequently found in the cardiac muscle which can lead to intracardiac thrombi [9]. In a retrospective study of 156 patients with cardiac amyloidosis who underwent transesophageal echocardiogram (TEE), intracardiac thrombi were identified in 27% [5].

There is often difficulty confirming the origin of these left atrial densities. Our case highlights the mimicry of cardiac thrombi in clinical presentation and appearance to a myxoid mass seen on an echocardiogram. The left atrium is the most common site of intra-atrial thrombus formation due to the anatomy of the left atrial appendage providing a blood stasis environment [10]. If the thrombus has well-defined borders with a stalk attached to the atrial septal wall, similar to our case, it may further increase suspicion of an atrial myxoma [4,11]. Although the management of an atrial mass should be based on the clinical situation (accompanying heart disease, etc.) and the echocardiography findings (well-limited mass, echogenicity, etc.), the distinction between myxomas and thrombi may pose considerable diagnostic challenges [11]. An intracardiac thrombus commonly presents as a mass in the left atrial appendage and may be associated with atrial fibrillation, atrial enlargement, mitral stenosis, or tricuspid stenosis [4]. With this presentation, the next step in management is anticoagulation to prevent these thrombi from causing ischemic complications when embolized. In some cases, surgical intervention is needed to remove the mass and obtain a biopsy of the collected sample to confirm the thrombolytic nature versus a myxoma. But atrial thrombi misdiagnosed as myxomas can lead to an unnecessary surgical resection [12].

Currently, there are no definitive guidelines on the anticoagulation needed or the duration required for the resolution of cardiac thrombi secondary to amyloidosis. However, there have been several studies on treatment modalities. It is possible that early detection of amyloidosis, vigilant screening for intracardiac thrombosis with early anticoagulation might improve the prognosis [13]. Effective anticoagulation might reduce thromboembolism, which is a significant contributor to mortality in cardiac amyloid patients [14]. 

There have been several studies comparing the various methods of anticoagulation therapy guided toward cardiac thrombi, however, these studies have focused on patients with underlying atrial fibrillation. For example, a TEE study used warfarin 2 mg daily for four to nine weeks showing resolution of the cardiac thrombi in 81.8% of patients with nonvalvular atrial fibrillation [10]. A CLOT-AF registry evaluated rivaroxaban 20/15 mg daily for six to eight weeks and showed a resolution rate of 62.5% of cardiac thrombi [10]. These studies and case reports used either warfarin or the direct oral anticoagulants (DOACs), which are now the mainstay of treatment options in patients with cardiac thrombi in the setting of atrial fibrillation [15]. However, there is still pending research to specifically address anticoagulation in patients with cardiac thrombi secondary to amyloidosis. Therefore, prospective studies are needed before definite recommendations can be made for patients similar to our case with cardiac thrombi without underlying atrial fibrillation. Overall, surgical resection, when feasible, is the treatment of choice for all benign cardiac densities. Surgical resection of myxomas and benign non-myxomal masses can lead to complete resolution of the patient’s symptoms. 

## Conclusions

This case explores the challenges in the diagnosis and management of intra-atrial thrombi with cardiac amyloidosis, commonly seen in the elderly population. Densities found on echocardiograms may presumptively be diagnosed as myxomas; however, other pathologies may mimic their clinical presentations, such as atrial thrombi. Various studies have explored the use of anticoagulation in patients with atrial thrombi secondary to atrial fibrillation or valvular diseases, but further studies are needed to address anticoagulation specifically for atrial thrombi secondary to amyloidosis. Given the diversity of etiologies, TEE and cardiac MRI may be helpful, but biopsy and histopathology of intracardiac masses are the golden standards of diagnosis. In conclusion, in agreement with the previous case reports, our case confirms the need for prospective studies addressing the management of cardiac amyloidosis in patients with a thrombotic presentation.
